# Computed Tomographic Imaging Features of COVID-19 Pneumonia Caused by the Delta (B.1.617.2) and Omicron (B.1.1.529) Variant in a German Nested Cohort Pilot Study Group

**DOI:** 10.3390/tomography8050202

**Published:** 2022-09-28

**Authors:** Esther Askani, Katharina Mueller-Peltzer, Julian Madrid, Marvin Knoke, Dunja Hasic, Fabian Bamberg, Christopher L. Schlett, Prerana Agarwal

**Affiliations:** 1 Medical Center, Department of Diagnostic and Interventional Radiology, University of Freiburg, 79106 Freiburg, Germany; 2 Pneumology, Angiology and Intensive Care, Department of Cardiology, Ortenau Klinikum, 77933 Lahr, Germany; 3 Faculty of Theology, Department of Protestant Theology, University of Heidelberg, 69117 Heidelberg, Germany

**Keywords:** COVID-19, Delta, Omicron, chest CT, lung involvement, SARS-CoV-2

## Abstract

Background: The aim of this study was to evaluate CT (computed tomography) imaging differences for the Delta and the Omicron variant in COVID-19 infection. Methods: The study population was derived from a retrospective study cohort investigating chest CT imaging patterns in vaccinated and nonvaccinated COVID-19 patients. CT imaging patterns of COVID-19 infection were evaluated by qualitative and semiquantitative scoring systems, as well as imaging pattern analysis. Results: A total of 60 patients (70.00% male, 62.53 ± 17.3 years, Delta: 43 patients, Omicron: 17 patients) were included. Qualitative scoring systems showed a significant correlation with virus variants; “typical appearance” and “very high” degrees of suspicion were detected more often in patients with Delta (RSNA: *p* = 0.003; CO-RADS: *p* = 0.002; COV-RADS: *p* = 0.001). Semiquantitative assessment of lung changes revealed a significant association with virus variants in univariate (Delta: 6.3 ± 3.5; Omicron: 3.12 ± 3.2; *p* = 0.002) and multivariate analysis. The vacuolar sign was significantly associated with the Delta variant (OR: 14.74, 95% CI: [2.32; 2094.7], *p* = 0.017). Conclusion: The Delta variant had significantly more extensive lung involvement and showed changes classified as “typical” more often than the Omicron variant, while the Omicron variant was more likely associated with CT findings such as “absence of pulmonary changes”. A significant correlation between the Delta variant and the vacuolar sign was observed.

## 1. Introduction

On 11 March 2020, COVID-19 was characterized as a pandemic by the World Health Organization (WHO) [1]. The coronavirus disease (COVID-19) is caused by SARS-CoV-2, which can provoke symptoms of severe viral pneumonia such as fever, cough, shortness of breath, and bilateral pulmonary infiltrates [2,3]. To date (as of August 2022), more than 590 million infections with COVID-19 have been recorded worldwide and more than 6 million deaths were attributed to COVID-19 [4]. In order to control the global spread of COVID-19, throughout 2020 multiple vaccines against COVID-19 were developed and became available at the end of 2020 [5]. However, since the beginning of the pandemic, new challenges have arisen due to the emergence of virus variants through the accumulation of mutations [6]. In late 2020, the WHO prompted the classification of novel SARS-CoV-2 strains as variants of interest (VOIs) and variants of concern (VOCs) [7]. The classification VOI refers to variants with mutations that result in changes to receptor binding, reduced efficacy of treatments, decreased neutralization by antibodies, and a potential increase in disease severity and/or transmissibility. On the other hand, the classification VOC refers to variants against which there may be strong evidence of an increase in transmissibility, greater disease severity, notable reduction in neutralization by antibodies generated, and thus decreased response to treatments and vaccines [8]. The two latest VOCs are the Delta (B.1.617.2) and Omicron (B.1.1.529) variants [9,10]. First detected in India, the Delta variant became the globally dominant strain in June 2021. Its mutations cause high transmissibility and less responsiveness to treatment, including vaccination, leading to a higher likelihood of severe cases, hospitalization, and deaths [11]. The Omicron variant followed the Delta variant as a globally dominant strain and was first detected in South Africa in November 2021. It is also characterized by high transmissibility; however, compared to the Delta variant, it is considered as less virulent and causes severe cases of disease, hospitalizations, and deaths at a lower rate than the Delta variant [12,13,14].

Chest CT plays a crucial role in COVID-19 infection diagnostics [15], although real-time reverse transcriptase polymerase chain reaction (RT-PCR) testing is considered the gold standard for the diagnosis of COVID-19 infection [16]. However, especially in the early phase of the pandemic, CT examination has played an important role for the triage at emergency departments, when the use of RT-PCR was limited because of logistical issues such as the development, mass production, and distribution of the examination kit [17]. In addition, in some cases, interpretation of chest CT can accelerate diagnosis. The radiologist may express a suspicion of COVID-19 due to typical imaging patterns on chest CT, as well as evaluate complications and potential differential diagnoses through chest CT [18,19,20]. However, data on CT findings of COVID-19 pneumonia originate mainly from early 2020 [21], before the Delta and Omicron variants appeared and became VOCs.

Only two recently published studies from Korea and the United Kingdom have accurately evaluated the potential differences in imaging patterns on chest CT between the Delta and Omicron variants. Soon et al. found that the Omicron SARS-COV-2 variant showed nontypical, peribronchovascular pneumonia and less pulmonary vascular involvement than the Delta variant in hospitalized patients with comparable CT disease severity [22]. Tsakok et al. found that patients with Omicron had lower median chest CT severity scores compared with Delta [23]. Overall, data on potentially different CT imaging patterns of COVID-19 due to emerging virus variants are scarce.

This study aimed to evaluate qualitative and semiquantitative chest CT imaging patterns in vaccinated and nonvaccinated hospitalized COVID-19 patients in a German pilot-study group and assess CT imaging differences for the Delta and Omicron variants.

## 2. Materials and Methods

### 2.1. Study Design and Study Population

The study population of this nested cohort study was derived from a retrospective single-center study cohort investigating vaccinated and nonvaccinated COVID-19 patients who received a chest CT performed within the study time window between 1 July 2021 and 14 February 2022 at the University Hospital of Freiburg (data not yet published). This study initially included patients with COVID-19 infection confirmed with at least one positive RT-PCR test (nasal or throat swab), and at least one chest CT examination during hospitalization. Patients with missing data on vaccination status, partial vaccination status, acute respiratory distress syndrome (ARDS), and age under 18 years were excluded. Additionally, patients with missing data on the COVID-19 virus variant were excluded for the population of this nested cohort study.

The Institutional Research Ethics Board of the Medical Faculty of the Albert-Ludwig-University Freiburg approved the retrospective single-center cohort study (22-1046-retro). The requirement for informed consent was waived due to the study’s retrospective nature. The requirements of the Helsinki Declaration on human research were met.

### 2.2. Vaccination Status

Vaccination status was divided into three categories: nonvaccinated, partially vaccinated, and fully vaccinated. Patients with a positive COVID-19 RT-PCR test result and onset of symptoms at least 14 days after receipt of the second vaccine dose (ChAdOx1 nCoV-19 vaccine-AstraZeneca, BNT162b2vaccine-Pfizer-BioNTech, mRNA-1273 vaccine–Moderna) were defined as fully vaccinated. Patients who had received Ad26.COV2.S vaccine-Johnson & Johnson-Janssen as the first vaccine required a second vaccine dose with a mRNA vaccine at least 14 days before a positive COVID-19 RT-PCR test result and onset of symptoms to be considered as fully vaccinated, as recommended in Germany by the Federal ministry of health [24]. The date of vaccination was not consistently recorded and could not be included.

### 2.3. Data Collection—Demographic and Clinical Laboratory Parameters

The original study cohort of consecutive patients was retrospectively identified in the electronic hospital information system and the radiological information system of the University Hospital of Freiburg. Following demographic and clinical laboratory data were extracted from electronic patient records: general information (age and sex), vaccination status, symptom onset, symptoms (dyspnea, cough, fever), pre-existing conditions (BMI, pre-existing diseases), treatment of COVID-19 infection (every kind of oxygen therapy such as noninvasive ventilation, high flow oxygen therapy, and intubation; intensive care treatment), complications (pulmonary superinfection, pulmonary artery embolism, exitus letalis), and virus variant. Data on COVID-19 reinfections were not systematically recorded and could not be included.

### 2.4. CT Examination

This study cohort’s hospitalized patients received a CT examination in case of therapeutic consequences, e.g., suspected pulmonary artery embolism, or in case of an unclear clinical constellation at the time of hospital admission. In all cases, a high-resolution chest CT (HRCT) was performed using the same Siemens Somatom Definition Flash (Siemens Healthineers, Erlangen, Germany). This study did not evaluate follow-up CT; only the first acquired CT scan following patient presentation was analyzed.

### 2.5. Radiological Analysis

CT scans were analyzed on axial reconstructed images with 1 mm slice thickness in lung window (W:1500 L:-600) on the Picture Archiving and Communication System (PACS—Dedalus HealthCare, Deep Unity Server 2.18.1, Deep Unity Diagnost 1.1.0.1 (client version)) by two independent readers (PA, 8 years of experience and EA, 3 years of experience) who were blinded to clinical data, vaccination status, virus variant, and stage of infection. For qualitative analysis, RSNA, CO-RADS, and COV-RADS scoring of the pneumonia was performed. For the classification based on the RSNA Expert Consensus Statement, the findings were classified as typical, indeterminate, atypical, and negative for pneumonia [21]. For the CO-RADS and COV-RADS classification, the level of suspicion of COVID-19 pneumonia from very low or category 1 up to very high or category 5 were scored [25,26]. The predominant pattern of pneumonia (ground glass opacity (GGO), consolidation, mixed, or fibrotic) was analyzed and additional note was made of the morphology of these features (rounded, subpleural, nonrounded nonsubpleural). Various components including crazy paving, reticulation, bronchiectasis, bronchial wall thickening, nodules, cavitation, lobar pneumonia, bronchoaerogram, vacuolar sign [27], organizing pneumonia, reverse halo sign, emphysema, and coronary calcification were scored. Additionally, the extent of lung involvement (single-lobe, unilateral, multilobar, bilateral) and the axial and craniocaudal distribution of the pneumonia were scored. The extent of pneumonia, GGO, and consolidation was also semiquantitatively scored per lung lobe (1 = less then 1/3, 2 = 1/3 − 2/3, 3 ≥ 2/3, maximum possible score of 15).

### 2.6. Inter- and Intrareader Variability

Inter- and intrareader variability were assessed in a random subset of 30 participants of the original study cohort. To avoid recall bias, measurements were performed with a time interval of at least 3 months. Inter- and intrareader variability were evaluated for the RSNA score, CO-RAD score, COV-RAD score, and for the semiquantitative scoring of CT-graphic pulmonary manifestations.

### 2.7. Statistical Analysis

Demographic and clinical parameters as well as qualitative and semiquantitative data of chest CT evaluation according to virus variants are presented as an arithmetic mean and standard deviation (SD) for continuous variables and as counts and percentages for categorical variables. Differences in continuous variables were evaluated by *t*-test, and Fisher’s exact test assessed differences in categorical variables. When necessary, QQ plots were used to test for normality, along with Levene’s tests to test for the homogeneity of variances, correlation coefficients to check for multicollinearity, a visual check for linearity of independent variables, and log odds and Cook’s distance to check for strongly influential outliers. Because participants were randomly selected, independence was assumed. Failure to fulfill parametric assumptions led to the use of Wilcoxon rank-sum and signed-rank tests. To determine associations of COVID-19 variants with semiquantitative lung involvement, a multiple logistic regression with outcome CT-graphic pulmonary manifestations was calculated with stepwise adjustment. Model 1 was adjusted for vaccination status; Model 2 was additionally adjusted for stage of infection (early stage: CT scan 0–5 days after symptom onset; progressive stage: CT scan 5–8 days after symptom onset; peak stage: 9–13 days after symptom onset; late stage: ≥14 days after symptom onset). Odds ratios (OR) with corresponding 95% confidence intervals (CI) were calculated per standard deviation (SD) of the variable of interest to enable comparability between effect estimates. To assess inter- and intrareader variability, Krippendorff’s alpha reliability estimate was used. Acceptable reliability was indicated by Krippendorff’s alpha value ≥ 0.667, high reliability was indicated by Krippendorff’s alpha ≥ 0.800. All analyses were conducted with R 4.2.0 [28]. *p*-values < 0.05 were considered to denote statistical significance.

## 3. Results

Initially, 105 patients were included in the analysis to assess CT imaging differences of COVID-19 pneumonia in vaccinated and nonvaccinated patients. In 60 patients (70.00% male, 62.53 ± 17.3 years), the virus variant was tested; these were therefore included in the analysis of the present study. In 43 cases, the Delta (B.1.617.2) variant was detected, and in 17 cases the Omicron (B.1.1.529) variant was detected. Patient demographics according to COVID-19 variant are presented in Table 1.

### 3.1. Clinical Parameters According to Virus Variants

A total of 34 of 60 patients (56.66%) were fully vaccinated. Vaccination status showed a significant correlation with virus variant in univariate analysis: while only 46.51% of patients infected with the Delta (B.1.617.2) variant were vaccinated, 82.38% of patients infected with the Omicron (B.1.1.529) variant were vaccinated (*p* = 0.019). Furthermore, in univariate analysis, pulmonary artery embolism (Delta: 2/43 (5.0%); Omicron: 5/17 (29.41%); *p* = 0.020) was significantly correlated with the virus variant. No significant associations were observed between virus variants and age, sex, pre-existing conditions, or necessitated COVID-19 therapy. Participants’ clinical data are presented in Table 1.

### 3.2. Qualitative Scoring, Pattern Distribution, Morphology, and Virus Variant

**Figure 1:**
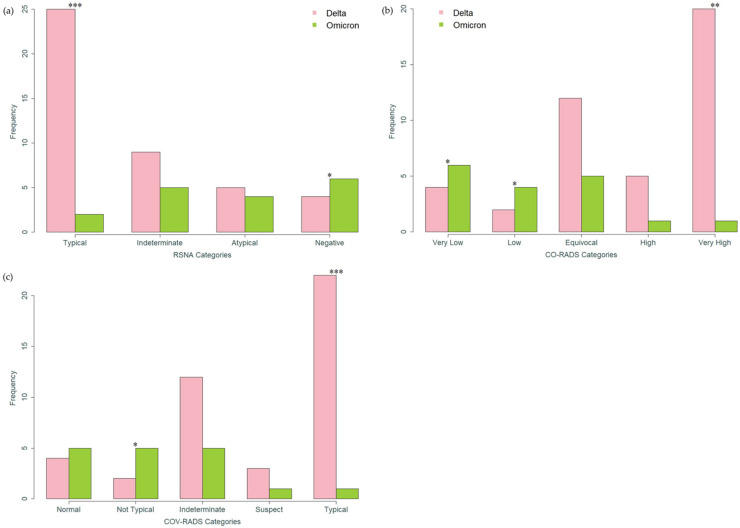
Bar charts with *p*-values from Fisher’s exact test. (**a**) Distribution of COVID-19 radiological RSNA scoring system according to virus variant. (**b**) Distribution of COVID-19 radiological CO-RADS scoring system according to virus variant. (**c**) Distribution of COVID-19 radiological COV-RADS scoring system according to virus variant. * (*p* < 0.05), ** (*p* < 0.01), *** (*p* ≤ 0.001) (see Table 2).

In univariate analysis, scoring systems for assessment of the probability of the presence of COVID-19 infection showed a significant correlation with virus variants (Table 2, Figure 1), whereby “typical appearance” and “very high” degree of suspicion were detected more often in patients infected with the Delta variant (RSNA: *p* = 0.001; CO-RADS: *p* = 0.003; COV-RADS: *p* = 0.001), and “absence of pneumonia”, “very low suspicion”, and “changes not typical for Covid” were detected more often in patients with the Omicron variant (RSNA: *p* = 0.024; CO-RADS: *p* = 0.024; COV-RADS: *p* = 0.016).

In multivariate analysis, after adjustment for vaccination status and stage of infection, these correlations remained significant in the categories “typical appearance” (RSNA), “very high” degree of suspicion (CO-RADS), and “typical” (COV-RADS) with outcome “categories” with an 8-, 9- and 11-fold higher odds for the Delta variant, respectively (Model 2: RSNA: OR: 8.08, 95% CI: [1.58; 63.94], *p* = 0.021; CO-RADS: OR: 9.32, 95% CI: [1.44; 184.47], *p* = 0.047; COV-RADS: OR: 10.91, 95% CI: [1.58; 225.43], *p* = 0.039). In addition, after adjustment for vaccination status and stage of infection, the categories “negative for pneumonia” (RSNA) and “very low” degree of suspicion (CO-RADS) remained significantly correlated with the virus variant with 6-fold higher odds for the Omicron variant, respectively (RSNA: OR: 5.66, 95% CI: [1.12; 35.41], *p* = 0.043; CO-RADS: 5.66, 95% CI: [1.12; 35.41], *p* = 0.043). Only the initially significant correlation between virus variant and the category “pathological, but not typical for Covid” (COV-RADS) became nonsignificant in multivariate analysis after adjustment for stage of infection (Table 3).

Furthermore, in univariate analysis, significant correlations between virus variant and pattern distribution as well as morphology were observed for: “lung involvement” (*p* = 0.04127), with higher rate of “no lung involvement” for Omicron (6/17 (35.29%)) than for Delta (5/43 (11.63%)) and a higher rate of “bilateral lung involvement” for Delta (36/43 (83.72%)) than for Omicron (10/17 (58.82%)); “distribution and pattern predominance”, with higher rate of “peripheral distribution” for Delta than for Omicron (Delta: 23/43 (53.49%), Omicron: 4/17 (23.53%), *p* = 0.046); “craniocaudal distribution”, with a higher rate of “no predominant distribution” for Omicron than for Delta (Delta: 4/43 (9.3%), Omicron: 6/17 (35.29%), *p* = 0.024); “GGO morphology”, with a higher rate of “subpleural distribution” for Delta than for Omicron (Delta: 25/43 (58.14%), Omicron: 3/17 (17.65%), *p* = 0.009); and “GGO/consolidation morphology”, with a higher rate of “GGO/consolidation absence” for Omicron than for Delta (Delta: 4/43 (9.3%), Omicron: 6/17 (35.29%), *p* = 0.024).

Moreover, in univariate analysis, crazy paving was significantly associated with the Delta variant (Delta: 14/43 (32.56%), Omicron: 1/17 (5.88%), *p* = 0.046), and the vacuolar sign was significantly associated with the Delta variant (Delta: 23/43 (53.49%), Omicron: 1/17 (5.88%), *p* = 0.001) (Table 2).

**Figure 2:**
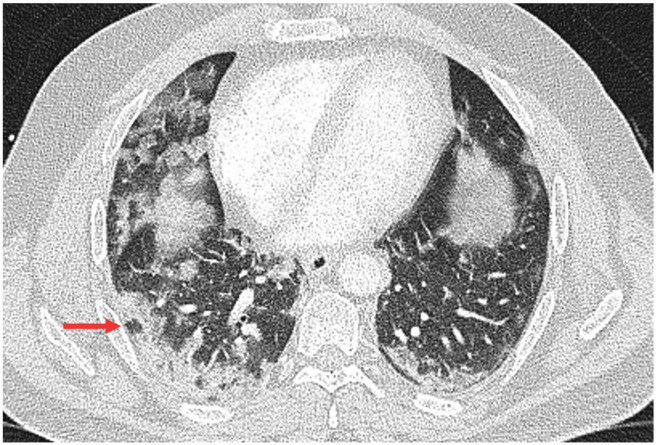
Vacuolar sign (red arrow) in right lower lobe in a 44-year-old male patient with the Delta variant COVID-19 infection.

In multiple logistic regression analysis, the crazy-paving pattern no longer showed a significant correlation with the virus variant after adjustment for vaccination status. In contrast, the vacuolar sign persistently showed a significant correlation with virus variant after adjustment for vaccination status and stage of infection with 14-fold higher odds of presence of the vacuolar sign with the Delta variant (Delta: OR: 14.74, 95% CI: [2.32; 2094.7], *p* = 0.017) (Table 3). An example of the vacuolar sign is provided in Figure 2.

### 3.3. Semiquantitative Scoring and Virus Variant

**Figure 3:**
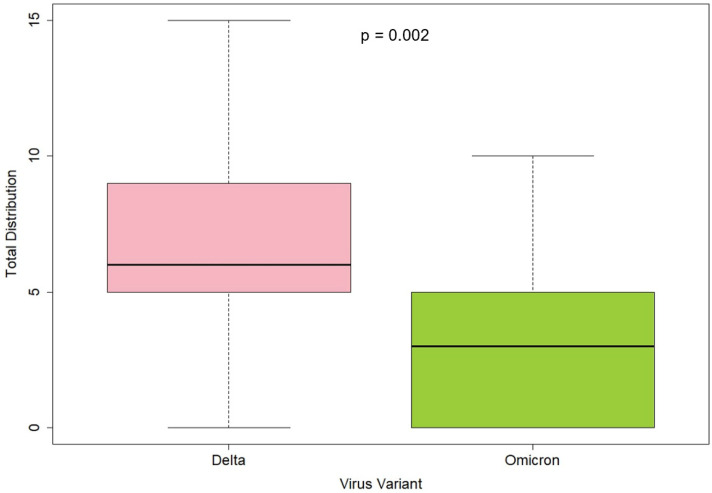
Box and whisker plot. Semiquantitative total distribution according to virus variant. *p* = 0.002 from *t*-test.

The semiquantitative assessment of lung changes due to COVID-19 infection revealed a significant association between virus variant and “total distribution” in univariate analysis (Delta: 6.3 ± 3.5; Omicron: 3.12 ± 3.2; *p* = 0.002); absence of pulmonary manifestation was especially significantly correlated to virus variant (absence in right upper lobe: Delta 8/43 (18.6%), Omicron 12/17 (70.59%), *p* = 0.0002; absence in right middle lobe: Delta 10/43 (23.26%), Omicron 10/17 (58.82%), *p* = 0.014; absence in right lower lobe: Delta 6/43 (13.95%), Omicron 7/17 (41.18%), *p* = 0.035; absence in left lower lobe: Delta 6/43 (13.95%), Omicron 8/17 (47.06%), *p* = 0.015). In addition, semiquantitative scoring for ground glass opacity (GGO) and consolidation was significantly correlated to virus variant (GGO: Delta 4.23 ± 3.01, Omicron 2.12 ± 2.47, *p* = 0.017; consolidation: Delta 2.49 ± 2.54, Omicron 1.06 ± 1.48, *p* = 0.034) (Table 4, Figure 3).

**Figure 4:**
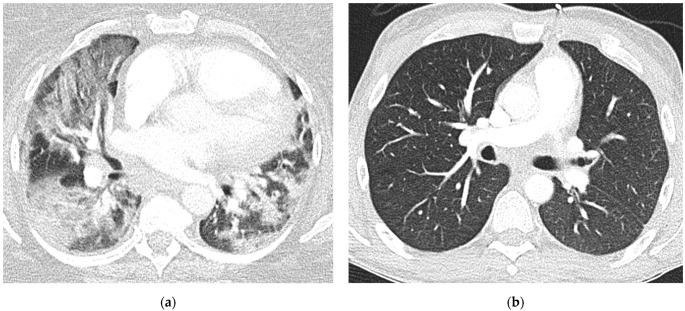
(**a**) A 54-year-old female patient with Delta variant with extensive lung involvement (semiquantitative score “15”); (**b**) a 40-year-old male patient with Omicron variant with “absence of pneumonia” (semiquantitative score “0”).

In multiple logistic regression, adjustment for vaccination status and stage of infection showed a persisting, significant association between virus variant and semiquantitative assessment of total distribution (Estimate β: 2.73, 95%CI: [0.75, 4.71]; *p* = 0.008), while associations between virus variant and GGO scoring as well as consolidation scoring were attenuated and became nonsignificant (Table 5). Examples for semiquantitative image analysis are provided in Figure 4.

### 3.4. Inter- and Intrareader Variability

For all radiological scoring systems as well as for semiquantitative assessment, inter- and intrareader variability showed high reliability (Krippendorff’s alpha coefficient for interreader variability: RSNA score with α = 0.891, CO-RADS with α = 0.800, COV-RADS with α = 0.842, “total distribution” with α = 0.831; Krippendorff’s alpha coefficient for intrareader variability: RSNA score with α = 0.893, CO-RADS with α = 0.804, COV-RADS with α = 0.849, “total distribution” with α = 0.94).

## 4. Discussion

Chest CT plays a key role in COVID-19 infection diagnostics [15]. However, little is known about different imaging patterns in chest CT regarding changing virus variants and progress of vaccination coverage. The two latest VOCs are the Delta (B.1.617.2) and Omicron (B.1.1.529) variants [9,10]. Studies published to date that have evaluated this topic show partially conflicting results [22,23].

In this German pilot study cohort, we studied 60 vaccinated and nonvaccinated COVID-19 patients (70.00% male, 62.53 ± 17.3 years). In 43 patients the Delta variant was detected, and in 17 patients the Omicron variant was detected. We found that the virus variant had a significant association with specific categories from the RSNA, CO-RADS, and COV-RADS scoring systems regardless of vaccination status or stage of infection. While the Delta variant was significantly more frequently associated with the categories “typical” and “very high suspicion”, Omicron was significantly more frequently associated with the categories “absence of pneumonia”, “very low suspicion”, and “changes not typical for pneumonia”. Semiquantitative evaluation showed that the extent of lung involvement was significantly associated with the virus variant. Delta showed a substantially more extensive lung involvement than Omicron.

Overall, our data support the notion that infections with Omicron appear to provoke less extensive parenchymal changes. The Omicron variant is regarded as less virulent regarding the rate of hospitalization, intensive care unit admissions, and mortality [12,13,14]. As far as we know, this is the first study observing a significant correlation between virus variant and the vacuolar sign, with a 14-fold higher probability of vacuolar sign in patients with the Delta variant. From a radiologist’s point of view, alertness is required when reporting the occurrence of nontypical changes in relation to COVID-19 infections. Moreover, given our findings, chest CT’s role in the COVID-19 pandemic may need to be reevaluated.

Our results are primarily in line with Tsakok et al., who found that in patients infected with the Omicron variant, more CT pulmonary angiograms were categorized as normal than those infected with the Delta variant [23]. Interestingly, our results on the extent of lung involvement, and the results of Tsakok et al., are contrary to the results of Yoon et al., who found that pneumonia extent and volume were not different between the variants after adjustment for confounders of age, comorbidities, vaccination, and infection duration [22]. A possible explanation for these differing results could be the different underlying indications for chest CT acquisition. While in Yoon et al.’s study, all patients received a baseline CT after admission, in our study, COVID-19 patients only received a chest CT in case of clinical relevance or therapeutic consequence. To reduce potential confounders, we adjusted for the stage of infection, and results on the extent of lung involvement remained significantly associated with the virus variant.

It has been described that Omicron replicates better in the bronchi and worse in the lung parenchyma [29]. This could explain the observed minor lung involvement and the minor rate of peripheral distribution of lung manifestation during COVID-19 infection in patients with the Omicron variant. Additionally, this may explain the minor rate of observed “typical appearance” in patients with the Omicron variant, or—like in Yoon et al.’s study—the observed peribronchovascular predilection in chest CT of the Omicron variant. Tsakok et al. also found that bronchial wall thickening was more common with the Omicron than with the Delta variant, which matched the findings in our study population: bronchial wall thickening was more frequently observed for the Omicron variant than for the Delta variant (Delta: 9/43 (20.93%), Omicron: 7/17 (41.18%), *p* = 0.193); however, correlations were not significant.

Furthermore, Yoon et al. observed that only 32% of patients with the Omicron SARS-CoV-2 variant had typical GGO at CT versus 57% of those with the Delta variant. In our case, the examinations also revealed that patients with Delta presented with more subpleural GGO on chest CT, and GGO scoring was significantly higher for patients with the Delta variant than those with the Omicron variant. Similarly, consolidation scoring was considerably higher for the Delta variant than for the Omicron variant. These last two observations may simply reflect the fact that Omicron’s lung manifestation is lower.

In our study, the vacuolar sign, a small air-containing space <5 mm in length within the lung lesion [27], was detected to be significantly associated with the Delta variant. Zhou et al. considered the vacuolar sign an indication of progressive disease [27]. However, in our study, associations remained significant after adjustment for the stage of infection.

In patients with the Delta variant, “typical chest CT appearance” of COVID-19 was detected to a significantly higher degree than in patients with the Omicron variant. This leads to the assumption that CT characteristics of COVID-19 may change with time due to emerging virus variants, which may lead to misinterpretations of chest CTs and delayed diagnoses of COVID-19. Altogether, constant evaluation and monitoring of variables influencing chest CT appearances of COVID-19 and analysis of their consequences for imaging patterns remain of great significance.

A few limitations of our study warrant mentioning. First, our study cohort is very small. The intention of this study was to provide initial data in a small German cohort regarding chest CT imaging changes depending on COVID-19 variants. Second, a higher rate of reinfections has been described for Omicron [30], but COVID-19 reinfections were not systematically recorded in our study. Consequently, speculation about the influence of reinfections on the observed reduced extent of lung changes cannot be finally clarified. Third, there was no classification of the clinical severity of patients, for which data could have been adjusted. However, clinical data showed no significant difference between the necessity of oxygen therapy or intensive care. Fourth, more patients with the Omicron variant were vaccinated, which might also have influenced the extent of pneumonia manifestation. However, adjustment for vaccination status was performed in multivariate analysis. Fifth, only hospitalized COVID-19 patients were included in this study, leaving out mild or asymptomatic infections, so caution is advised concerning the generalizability of the results.

## 5. Conclusions

In conclusion, our results line up with observations that the Omicron variant causes less extensive parenchymal changes. We found that the Delta variant had significantly more extensive lung involvement than the Omicron variant. In addition, the Omicron variant was significantly more likely associated with CT findings such as “negative for pneumonia” and “absence of pulmonary changes”. The Delta variant showed significantly more changes classified as “typical”. To the best of our knowledge, this pilot study is the first study observing a significant correlation between the virus variant and the vacuolar sign, with a 14-fold higher probability of the vacuolar sign in COVID-19 patients infected with the Delta variant. Further studies are needed to investigate the role of CT imaging in pandemic change and the influence of future virus variants on CT imaging.

## Figures and Tables

**Table 1:** Patients’ demographic and clinical parameters according to virus variants.

		Virus Variant		
	All	Delta *(n = 43)*	Omicron *(n = 17)*	*p*-value
General Information				
age (years) *(n = 60)* sex *(n = 60)* male female	62.53 ± 17.3 42 (70.00%) 18 (30.00%)	61.79 ± 16.01 29 (67.44%) 14 (32.56%)	64.41 ± 20.63 13 (76.47%) 4 (23.53%)	0.601 * 0.550
vaccination status *(n = 60)*				
nonvaccinated vaccinated	26 (43.33%) 34 (56.66%)	23 (53.49%) 20 (46.51%)	3 (17.5%) 14 (82.38%)	0.019
Symptoms				
dyspnea *(n = 60)* cough *(n = 57)* fever *(n = 58)*	45 (75.00%) 29 (50.88%) 32 (55.17%)	34 (79.07%) 22 (55.00%) 24 (58.54%)	11 (64.71%) 7 (41.18%) 8 (47.06%)	0.324 0.395 0.563
Pre-existing Conditions				
BMI *(n = 52)*				
<25 kg/m^2^ ≥25 kg/m^2^ pre-existing disease *(n = 60)*	21 (40.38%) 31 (59.62%) 52 (86.67%)	14 (35.9%) 25 (64.1%) 36 (83.72%)	7 (53.85%) 6 (46.15%) 16 (94.12%)	0.333 0.420
Treatment of COVID-19-Infection				
oxygen therapy *(n = 53)* intensive therapy *(n = 60)*	40 (75.47%) 13 (21.67%)	31 (79.49%) 12 (27.91%)	9 (64.29%) 1 (5.88%)	0.292 0.086
Complications				
pulmonary superinfection *(n = 58)* pulmonary artery embolism *(n = 57)* exitus letalis *(n = 59)*	19 (32.76%) 7 (12.28%) 6 (10.16%)	15 (36.59%) 2 (5.00%) 5 (11.9%)	4 (23.53%) 5 (29.41%) 1 (5.88%)	0.378 0.020 0.662

For continuous variables, values are mean ± standard deviation (SD) with *p*-values from *t*-test (*). For categorical variables, values are counts and percentages with *p*-values from Fisher’s exact test. Abbreviations: BMI: body mass index.

**Table 2:** Qualitative-scoring CT-graphic pulmonary and thoracic manifestation of COVID-19 infection according to the virus variant.

		Virus Variant		
	All *(n = 60)*	Delta *(n = 43)*	Omicron *(n = 17)*	*p*-value
RSNA categories				0.003
typical appearance indeterminate appearance atypical appearance negative for pneumonia	27 (45.00%) 14 (23.33%) 9 (15.00%) 10 (16.67%)	25 (58.14%) 9 (20.93%) 5 (11.63%) 4 (9.30%)	2 (11.76%) 5 (29.41%) 4 (23.53%) 6 (35.29%)	0.001 0.511 0.256 0.024
CO-RADS				0.002
very low low equivocal high very high	10 (16.67%) 6 (10.00%) 17 (28.33%) 6 (10.00%) 21 (35.00%)	4 (9.30%) 2 (4.65%) 12 (27.91%) 5 (11.63%) 20 (46.51%)	6 (35.29%) 4 (23.53%) 5 (29.41%) 1 (5.88%) 1 (5.88%)	0.024 0.048 1 0.665 0.003
COV-RADS				0.001
normal lung pathological, but not typical for Covid indeterminate suspect of Covid typical	9 (15.00%) 7 (11.66%) 17 (28.33%) 4 (6.67%) 23 (38.34%)	4 (9.30%) 2 (4.65%) 12 (27.91%) 3 (6.98%) 22 (51.16%)	5 (29.41%) 5 (29.41%) 5 (29.41%) 1 (5.88%) 1 (5.88%)	0.101 0.016 1 1 0.001
Distribution and Pattern Predominance				
lung involvement				0.041
no lung involvement single lobe unilateral multilobar bilateral	11 (18.33%) 1 (1.67%) 2 (3.33%) 46 (76.67%)	5 (11.63%) 0 (0%) 2 (4.65%) 36 (83.72%)	6 (35.29%) 1 (5.88%) 0 (0%) 10 (58.82%)	0.059 0.283 1 0.087
axial distribution				0.053
no predominant distribution peripheral distribution central distribution diffuse distribution	11 (18.33%) 27 (45.00%) 4 (6.66%) 18 (30.00%)	5 (11.63%) 23 (53.49%) 2 (4.65%) 13 (30.23%)	6 (35.29%) 4 (23.53%) 2 (11.76%) 5 (29.41%)	0.059 0.046 0.317 1
craniocaudal distribution				0.080
no predominant distribution upper lobe predominant lower lobe predominant diffuse	10 (16.67%) 4 (6.67%) 18 (30.00%) 28 (46.67%)	4 (9.30%) 4 (9.30%) 13 (30.23%) 22 (51.16%)	6 (35.29%) 0 (0%) 5 (29.41%) 6 (35.29%)	0.024 0.570 1 0.390
Pattern Morphology				
GGO morphology GGO absent subpleural rounded nonrounded, nonperipheral	15 (25.00%) 28 (46.67%) 2 (3.33%) 15 (25.00%)	8 (18.60%) 25 (58.14%) 2 (4.65%) 8 (18.60%)	7 (41.18%) 3 (17.65%) 0 (0%) 7 (41.18%)	0.014 0.099 0.009 1 0.099
GGO/consolidation				0.049
morphology				
GGO/consolidation absent predominant extensive predominant nodular GGO/consolidation mixed	10 (16.67%) 38 (63.33%) 8 (13.33%) 4 (6.67%)	4 (9.30%) 30 (69.77%) 5 (11.63%) 4 (9.30%)	6 (35.29%) 8 (47.06%) 3 (17.65%) 0 (0%)	0.024 0.139 0.676 0.570
Other Pulmonary Findings				
crazy paving reticulation bronchiectasis bronchial wall thickening tree-in-bud bronchoaerogram vacuolar sign reverse halo sign COP pattern	15 (25%) 15 (25%) 10 (16.67%) 16 (26.67%) 5 (8.34%) 14 (23.34%) 24 (40.00%) 4 (6.67%) 13 (21.66%)	14 (32.56%) 10 (23.26%) 6 (13.95%) 9 (20.93%) 4 (9.3%) 13 (30.23%) 23 (53.49%) 4 (9.3%) 11 (25.58%)	1 (5.88%) 5 (29.41%) 4 (23.53%) 7 (41.18%) 1 (5.88%) 1 (5.88%) 1 (5.88%) 0 (0%) 2 (11.76%)	0.046 0.743 0.448 0.193 1 0.050 0.001 0.570 0.314

For categorical variables, values are counts and percentages with *p*-values from Fisher’s exact test. Abbreviations: GGO: ground glass opacity; COP: cryptogenic organizing pneumonia.

**Table 3:** Multiple logistic regression analysis of association of CT-graphic pulmonary manifestations and virus variant.

	Virus Variant				
OUTCOME	Delta		Omicron		
	OR	95%CI	OR	95%CI	*p*-value
Model 1: Association of CT-graphic pulmonary manifestations and virus variant, adjusted for vaccination status					
RSNA Categories					
*typical appearance* vs. *all other categories* *negative for pneumonia* vs. *all other categories*	8.25 0.15	[1.90, 57.93] [0.03, 0.71]	0.12 6.64	[0.02, 0.53] [1.41, 39.07]	0.011 0.022
CO-RADS Categories					
*very high* vs. *all other categories* *very low* vs. *all other categories*	10.98 0.15	[1.86, 210.25] [0.03, 0.71]	0.09 6.64	[0.00, 0.54] [1.41, 39.07]	0.028 0.022
COV-RADS Categories					
*typical* vs. *all other categories* *pathological, but not typical for Covid* vs. all other categories	11.72 0.16	[1.94, 226.52] [0.02, 0.89]	0.09 6.35	[0.00, 0.52] [1.12, 51.25]	0.025 0.048
crazy paving	4.41	[0.65; 87.78]	0.23	[0.01; 1.54]	0.190
vacuolar sign	15.5	[2.66; 296.64]	0.06	[0.003; 0.38]	0.012
Model 2: Model 1 + adjusted for stage of infection					
RSNA Categories					
*typical appearance* vs. *all other categories* *negative for pneumonia* vs. *all other categories*	8.08 0.18	[1.58, 63.94] [0.03, 0.89]	0.12 5.66	[0.02, 0.63] [1.12, 35.41]	0.021 0.043
CO-RADS Categories					
*very high* vs. *all other categories* *very low* vs. *all other categories*	9.32 0.18	[1.44, 184.47] [0.03, 0.89]	0.11 5.66	[0.01, 0.69] [1.12, 35.41]	0.047 0.043
COV-RADS Categories					
*typical* vs. *all other categories* *pathological, but not typical for Covid* vs. *all other categories*	10.91 0.22	[1.58, 225.43] [0.02, 1.47]	0.09 4.49	[0.00, 0.63] [0.68, 40.01]	0.039 0.132
crazy-paving	4.49	[0.65; 90.28]	0.22	[0.01; 1.55]	0.188
vacuolar sign	14.74	[2.32; 2094.7]	0.07	[0.003; 0.43]	0.017

Results of a logistic regression model with outcomes in pulmonary changes and exposure vaccination status. Abbreviations: OR—odds ratio; CI—confidence interval.

**Table 4:** Semiquantitative scoring: CT-graphic pulmonary manifestation of COVID-19 infection according to virus variant.

		Virus Variant		
	All *(n = 60)*	Delta *(n = 43)*	Omicron *(n = 17)*	*p*-value
Total Distribution				
semiquantitative scoring (mean ± SD)	5.4 ± 3.69	6.3 ± 3.5	3.12 ± 3.2	0.002 *
Distribution right upper lobe				0.002
absent	20 (33.33%)	8 (18.6%)	12 (70.59%)	0.0002
<1/3	26 (43.34%)	22 (51.16%)	4 (23.53%)	0.082
1/3–2/3	10 (16.67%)	9 (20.93%)	1 (5.88%)	0.255
>2/3	4 (6.67%)	4 (9.30%)	0 (0%)	0.570
Distribution right middle lobe				0.080
absent	20 (33.34%)	10 (23.26%)	10 (58.82%)	0.014
<1/3	27 (45.00%)	22 (51.16%)	5 (29.41%)	0.158
1/3–2/3	11 (18.33%)	9 (20.93%)	2 (11.76%)	0.712
>2/3	2 (3.33%)	2 (4.65%)	0 (0%)	1
Distribution right lower lobe				0.152
absent	13 (21.67%)	6 (13.95%)	7 (41.18%)	0.035
<1/3	25 (41.66%)	20 (46.51%)	5 (29.41%)	0.260
1/3–2/3	18 (30.00%)	14 (32.56%)	4 (23.53%)	0.550
>2/3	4 (6.67%)	3 (6.98%)	1 (5.88%)	1
Distribution left upper lobe				0.062
absent	17 (28.33%)	9 (20.93%)	8 (47.06%)	0.059
<1/3	21 (35.00%)	14 (32.56%)	7 (41.18%)	0.560
1/3–2/3	16 (26.66%)	14 (32.56%)	2 (11.76%)	0.120
>2/3	6 (10.00%)	6 (13.95%)	0 (0%)	0.170
Distribution left lower lobe				0.047
absent	14 (23.33%)	6 (13.95%)	8 (47.06%)	0.015
<1/3	29 (48.34%)	22 (51.16%)	7 (41.18%)	0.573
1/3–2/3	13 (21.66%)	11 (25.58%)	2 (11.76%)	0.314
>2/3	4 (6.67%)	4 (9.30%)	0 (0%)	0.570
GGO Scoring				
semiquantitative scoring (mean ± SD)	3.63 ± 3	4.23 ± 3.01	2.12 ± 2.47	0.017 **
Consolidation Scoring				
semiquantitative scoring (mean ± SD)	2.08 ± 2.37	2.49 ± 2.54	1.06 ± 1.48	0.034 **

For continuous variables, values are mean ± standard deviation (SD) with *p*-values from *t*-test (*) and from Mann–Whitney U test (Wilcoxon rank-sum test) (**), where appropriate. For categorical variables, values are counts and percentages with *p*-values from Fisher’s exact test. Abbreviation: GGO: ground glass opacity.

**Table 5:** Multiple regression analysis of semiquantitative lung involvement and virus variant, adjusted for vaccination status and stage of infection.

Predictor	Estimate (*β*)	95%CI	*p*-Value
Model 1: Association of semiquantitative lung involvement and virus variant, adjusted for vaccination status.			
Total Distribution			
Delta (REF: Omicron)	3.22	[1.13, 5.31]	0.003
GGO Scoring			
Delta (REF: Omicron)	2.08	[0.32, 3.84]	0.021
Consolidation Scoring			
Delta (REF: Omicron)	1.39	[−0.02, 2.79]	0.053
Model 2: Model 1 + adjusted for stage of infection.			
Total distribution			
Delta (REF: Omicron)	2.73	[0.75, 4.71]	0.008
GGO Scoring			
Delta (REF: Omicron)	1.48	[−1.15, 3.1]	0.074
Consolidation Scoring			
Delta (REF: Omicron)	1.37	[−0.04, 2.78]	0.057

Presented are results from multiple regression analysis with outcomes of semiquantitative lung involvement. Abbreviation: GGO: ground glass opacity.

## Data Availability

The data presented in this study are available on request from the corresponding author.
